# Surgical outcomes of robotic versus conventional autologous breast reconstruction: a systematic review and meta-analysis

**DOI:** 10.1007/s11701-024-01913-x

**Published:** 2024-05-02

**Authors:** Ali Mohamed Elameen, Asmaa Ali Dahy

**Affiliations:** 1 Department of Plastic and Reconstructive Surgery, El-Sahel Teaching Hospital, Cairo, Egypt; 2 Department of Plastic and Reconstructive Surgery, Faculty of Medicine For Girls, Al-Azhar University, Gameat Al Azhar, Nasr City, Cairo, Egypt

**Keywords:** Robotic, Autologous, Breast, Reconstruction

## Abstract

Breast reconstruction is an integral part of breast cancer management. Conventional techniques of flap harvesting for autologous breast reconstruction are associated with considerable complications. Robotic surgery has enabled a new spectrum of minimally invasive breast surgeries. The current systematic review and meta-analysis study was designed to retrieve the surgical and clinical outcomes of robotic versus conventional techniques for autologous breast reconstruction. An extensive systematic literature review was performed from inception to 25 April 2023. All clinical studies comparing the outcomes of robotic and conventional autologous breast reconstruction were included for meta-analysis. The present meta-analysis included seven articles consisting of 783 patients. Of them, 263 patients received robotic breast reconstruction, while 520 patients received conventional technique. Of note, 477 patients received latissimus dorsi flap (LDF) and 306 were subjected to deep inferior epigastric artery perforator (DIEP) flap. There was a significantly prolonged duration of surgery (MD 58.36;95% CI 32.05,84.67;*P* < 0.001) and duration of anaesthesia (MD 47;95% CI 16.23,77.77;*P* = 0.003) among patients who underwent robotic surgery. There was a similar risk of complications between robotic and conventional surgeries. The mean level of pain intensity was significantly lower among patients who received robotic breast surgery (MD− 0.28;95% CI − 0.73,0.17; *P* = 0.22). There was prolonged length of hospitalization among patients with conventional DIEP flap surgery (MD− 0.59;95% CI − 1.13,− 0.05;*P* = 0.03). The present meta-analysis highlighted the feasibility, safety, and effectiveness of robotic autologous breast reconstruction. This included the successful harvesting of LDF and DIEP flap with acceptable surgical and functional outcomes.

**Supplementary Information:**

The online version contains supplementary material available at 10.1007/s11701-024-01913-x.

## Introduction

Breasts are the symbolic expression of femininity, attractiveness, and motherhood. Breast cancer is the most diagnosed malignancy globally, accounting for nearly 12.5% of all recently recognized cancer patients. It is the second leading cause of cancer-related deaths in women worldwide [1]. Noteworthy, a considerable improvement in the management of patients with breast cancer has been noticed throughout the past era. This is attributed to greater awareness, early detection, and better therapeutic interventions [2, 3]. Mastectomy is considered a destructive experience resulting in substantial psychosexual repercussions. It changes the perception of body image, reducing self-esteem and psychological well-being [4, 5]. The increasing number of breast cancer survivors highlighted breast reconstruction's ultimate role in restoring the aesthetic appearance of breasts after mastectomies. Breast reconstruction could decrease the psychological burden of the disease, improving the sexual well-being and self-confidence among breast cancer survivors [6, 7].

Breast reconstruction is an integral part of breast cancer management. Breast reconstruction is categorized into either autologous or implant-based [8]. In the United States, nearly 19% of patients undergo autologous breast reconstruction yearly [9]. Whereby autologous breast reconstruction tends to be a more complex surgical procedure; it is associated with more desirable aesthetic and psychological outcomes [10]. The autologous breast reconstructive options commonly included abdominal-based flaps, latissimus dorsi flap (LDF), or free flaps [11]. Since the introduction of the deep inferior epigastric artery perforator (DIEP) flap, it has become the primary approach for autologous breast reconstruction. It is associated with minimal donor site complications and acceptable aesthetic outcomes for both abdomen and breasts [12] However, the DIEP flap may not be the ideal reconstructive option for patients with previous abdominal surgeries or those with inadequate abdominal tissue [13]. The LDF is a feasible alternative for such conditions. It restores the shape and function of the ptotic breasts and offers muscle coverage over breast implants [14].

Conventional open techniques of flap harvesting for breast reconstruction are associated with considerable complications. Conventional harvesting of the LDF can result in an apparent dorsal scar of 15 to 45 cm long. Conventional elevation of the DIEP flap necessitates a sizeable incision in the anterior rectus fascia to dissect the vascular pedicle [15, 16]. Extensive splitting, dissection, and traction of the anterior rectus fascia, motor nerves, and rectus muscle may result in significant donor site morbidity. This confers a high risk of abdominal wall herniation, motor weakness, bulging, and persistent post-operative pain [17, 18]. These consequences highlighted the need for more minimally invasive procedures to mitigate the potential shortcomings of conventional flap harvesting techniques.

Robotic technology may decrease the invasiveness during flap harvesting for autologous breast reconstruction [19, 20]. It is associated with better visualization, surgical dexterity, and cosmetic results in contrast to conventional techniques [21]. This decreases donor site complications and results in less post-operative pain and quick recovery. Paradoxically, robotic breast reconstruction may be associated with prolonged operation time and lesser flap volume and may necessitate a lengthy learning curve [22, 23]. Whereby the outcomes of robotic-based breast surgeries are promising, there is a continuous need for further evaluation of its surgical and clinical outcomes in the settings of breast reconstruction [24, 25]. Previously published systematic reviews are insufficient to draw conclusive evidence for current clinical practice. The results of these reviews are limited without a quantitative synthesis of the data. Understanding the merits and pitfalls of robotic autologous breast reconstruction can better aid surgeons in facilitating breast surgical care [26, 27]. Furthermore, there is a demanding concern to offer naturally looking and aesthetically pleasing breasts while minimizing donor site morbidity after breast reconstruction surgeries [28]. Therefore, the current systematic review and meta-analysis study was designed to retrieve the surgical and clinical outcomes of robotic versus conventional techniques for autologous breast reconstruction. This knowledge may provide a deeper insight into the areas for improvement for DIEP flap and LDF harvesting for autologous breast reconstruction.

## Materials and methods

The steps of the current meta-analysis study followed the guidelines and the recommendations offered through the Cochrane collaboration [29], and the Preferred Reporting Items for Systematic Reviews and Meta-Analysis (PRISMA) guidelines [30] (Supplementary Table 1). The methodology of the study was documented in the PROSPERO database (Number; CRD42023420626).

## Search methods

An extensive systematic literature review was performed from inception to 25 April 2023. Each database was searched using customized controlled vocabulary terms. A combination of medical subject heading, and text words were used to retrieve a wide range of potentially eligible articles. The systematic search included these databases; Google Scholar, PubMed, Web of Science (ISI), SIGLE, Scopus, NYAM, VHL, Controlled Trials (mRCT), Cochrane Collaboration, Clinical trials, WHO International Clinical Trials Registry Platform (ICTRP), and EMBASE. The following keywords were used; ‘Robot’, ‘Robotic’, ‘Robotics’, ‘Robotically’, ‘Reconstruction’, ‘Flap’,’ Flaps,’ Reconstructive’,’ Breast’. No restrictions were employed on patients’ age, sex, ethnicity, language, race, or place. Manual searching was performed to include all potentially relevant articles not retrieved throughout the searching of the databases. This included citation tracking, updated searching, cross-referencing, and screening of the citations of previous reviews.

### Study selection

All clinical studies comparing the outcomes of robotic and conventional autologous breast reconstruction were included for meta-analysis. Furthermore, non-comparative studies, review articles, studies with unextractable data, guidelines, cadaveric articles, case reports, erratum, letters, case series, comments, editorials, meeting abstracts, book chapters and posters were excluded. The title, abstract, and full-text screening were performed to disclose the potentially relevant articles that met the eligibility criteria. The PRISMA flowchart documented the searching process, screening, and the causes of articles exclusion at each step of the systematic literature review.

## Data extraction

The data were extracted in a well-organized Microsoft Excel sheet. The source-related data, including the title, study ID, study regions, study period, and study design, were extracted. The methods-related data were extracted, including the eligibility criteria, the robotic technique, the platform of the robot, the conventional technique, study endpoints, and follow-up periods. Baseline patients' demographic characteristics were extracted, including sample size, patients' age, body mass index (BMI), comorbidities, and previous history of abdominal surgeries. Breast cancer-related data were extracted to retrieve tumour pathology and stage of breast cancer. Breast surgery-related variables were revealed, including type of mastectomy, type of reconstruction, reconstruction timing, number of implants, and cup size. The outcomes of robotic surgery were shown, including duration of surgery, duration of anaesthesia, intra-operative blood loss, incision length, post-operative hospital stays, post-operative analgesics use, surgical complications, total costs, and satisfaction with breasts. The data reported only using graphs were extracted and converted using WebPlotDigitizer software [31].

## Quality assessment

The national institute of health (NIH) quality assessment tool was used to determine the quality of the included retrospective and prospective studies [32].

## Data analysis

Standardized mean difference (SMD) or weighted mean difference (WMD) was used for meta-analyzing the continuous data. Data reported using median and range was converted to mean and standard deviation (SD) based on Hozo et al. equations [33]. The risk ratio (RR) with a 95% confidence interval (CI) was used for analyzing binary variables. The fixed-effect model was used when homogeneity between the effect sizes was revealed. The random-effects model was used when the statistical heterogeneity was established. Statistical heterogeneity was determined using Higgins *I*^*2*^ statistic, at the value of > 50%, and the Cochrane Q (*Chi*^2^ test), at the value of *p* < 0.10 [34]. Review Manager version 5.4 and Comprehensive Meta-Analysis v3 software [35] were used to analyze the data. The significant difference between robotic and conventional techniques was revealed when the value of *P* < 0.05.

## Results

**Fig. 1:**
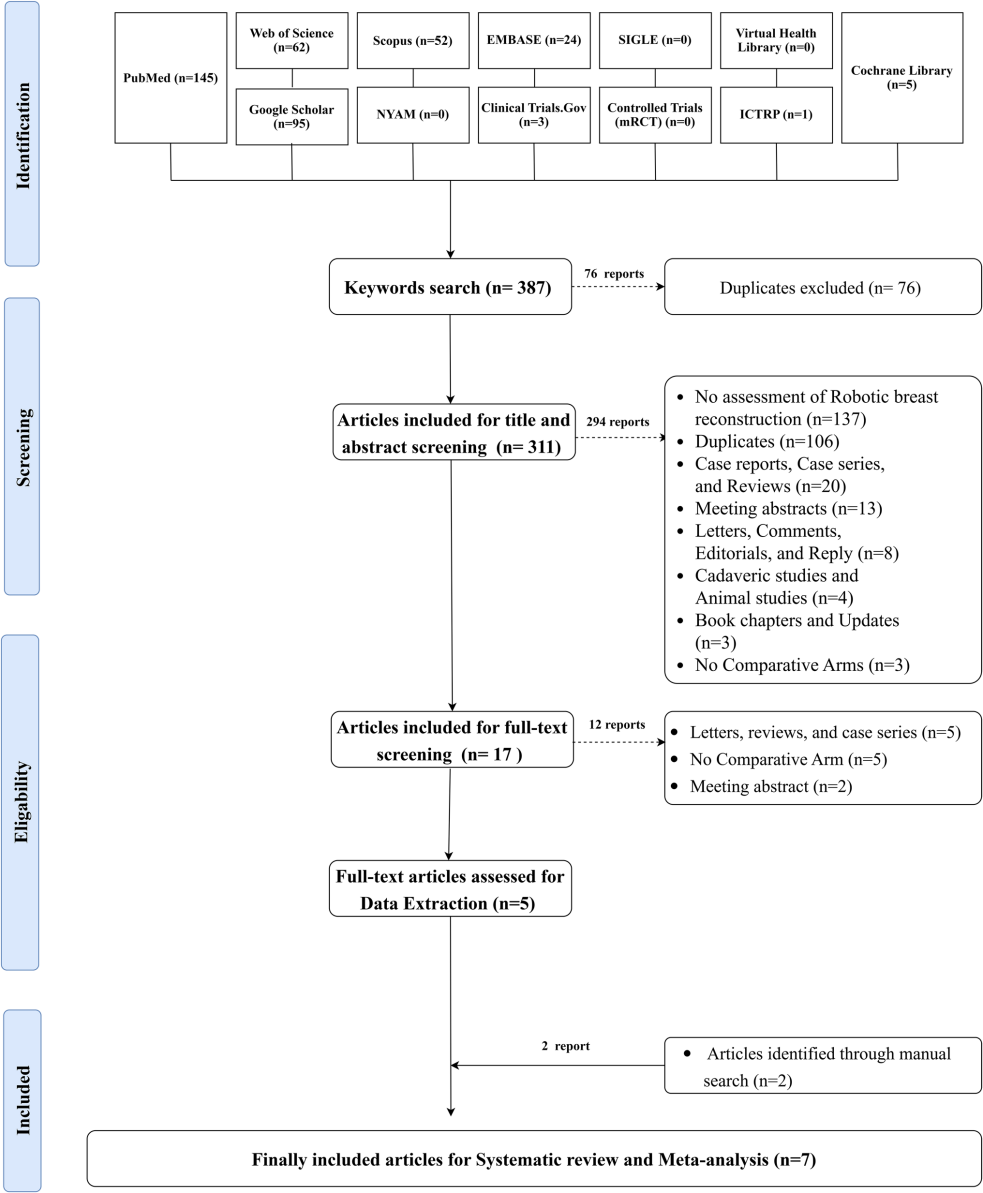
PRISMA Flow chart showing the process of the literature search, title, abstract, and full text screening, systematic review, and meta-analysis

A systematic search of the twelve databases resulted in 387 studies. Of them, 76 reports were excluded being duplicated, retrieving 311 articles eligible for title and abstract screening. The later process resulted in 17 articles being included for full-text screening. Twelve articles were ousted, resulting in five reports included for data extraction. Two articles were recognized throughout citation tracking and updated searching, resulting in seven articles finally being eligible for systematic review and meta-analysis. The process of searching databases, screening, and eligibility is shown in Fig. 1.

## Demographic characterestics of the included studies

The present meta-analysis included seven articles consisting of 783 patients [36–42]. Of them, 263 patients received robotic breast reconstruction, while 520 patients received conventional technique. There were five articles of prospective design, while two were retrospective. Of note, 477 patients received LDF and 306 were subjected to DIEP flap. Whereas robotic LDF was performed among 229 patients, conventional techniques were carried out among 248 patients. The robotic DIEP flap was performed among 34 patients using a transabdominal preperitoneal approach. Lee et al., 2022 used single port preperitoneal approach, while Tsai et al., 2023 used multiport robotic approach [40, 43]. The average age of the included patients ranged from 45.4 to 54.5 years and 45.6 to 56.1 years among robotic and conventional groups, respectively. Noteworthy, 103 patients received post-mastectomy radiotherapy among robotic surgery, and 165 patients within the conventional surgery group. The follow-up period ranged from 14.6 months to five years among the robotic surgery group and from 14 months to one year among the conventional surgery group (Table 1).

**Table 1:** Demographic characteristics of the included studies

Study ID		Study region	Registration number	Study design	Study period	Intervention	Platform of robotic surgery	Control	Sample size		Age (Years)	
									Robotic	Conventional	Robotic	Conventional
									Number	Number	Mean ± SD	Mean ± SD
1	Clemens et al. [36]	USA	NA	Retrospective	2009 and 2013	RALDF	da Vinci robot	Traditional open technique	12	64	54.3	56.1
2	Eo et al. [42]	South Korea	NA	Prospective case–control	March 2020 to December 2021	RALDF	da Vinci robotic surgical system	Conventional LDF	20	20	45.4 ± 5.7	46.6 ± 4.8
3	Houvenaeghel et al.[37]	France	NCT02869607	Prospective case–control	March 2016 and June 2019	RALDF	a Vinci Si® or Xi® surgical systems, Intuitive	Traditional latissimus dorsi flap	46	59	58.1	49.5
4	Houvenaeghel et al.[38]	France	NCT02869607)	Prospective case–control	January 2016 and July 2020	RALDF	da Vinci Si Ò surgical system XI or SI	Conventional LDF	126	78	54.5(52.94–57.44)*	50.5 (47.53–53.06)*
5	Lee et al., 2022[39]	South Korea	NR	Retrospective	July 2017 and January 2021	RA-DIEP Flap	da Vinci SP; intuitive surgical, Sunnyvale, CA	Conventional DIEP technique	21	186	48.5 ± 6.6	48.5 ± 7.8
6	Tsai et al. [40]	Taiwan	NR	Retrospective	May 2020 to May 2022	RA-DIEP Flap	da Vinci system, intuitive surgical Inc., Sunnyvale, CA	Conventional DIEP	13	86	46 ± 10.96	45.6 ± 7.24
7	Winocour et al. [41]	USA	NR	Retrospective	June of 2011 to June of 2015	RALDF	NR	Traditional LDF	25	27	51 ± 9.7	50 ± 8.7

Study ID		BMI (Kg/m^2^)		Comorbidities						Pre-operative radiotherapy		Follow-up period (months)	
				Diabetes mellitus		Hypertension		Current smoking					
		Robotic	Conventional	Robotic	Conventional	Robotic	Conventional	Robotic	Conventional	Robotic	Conventional	Robotic	Conventional
		Mean ± SD	Mean ± SD	Number	Number	Number	Number	Number	Number	Number	Number	Number	Number
1	Clemens et al. [36]	25.4	25.9	NR	NR	NR	NR	3	14	12	64	14.6 ± 7.3	16.4 ± 6.9
2	Eo et al. [42]	23.7 ± 3.3	22.8 ± 2.7	0	0	0	0	0	0	20	20	18.4 ± 4.6	18.4 ± 7.1
3	Houvenaeghel et al.[37]	25.7	24.1	NR	NR	3	0	10	11	5	11	NR	NR
4	Houvenaeghel et al.[38]	23.51 (24.04–25.69)*	23.7 (23.41–25.06)*	NR	NR	6	0	27	14	53	49	NR	NR
5	Lee et al., 2022[39]	23.9 ± 3.6	23.9 ± 3.0	2	21	0	9	0	3	NR	NR	Six	
6	Tsai et al. [40]	23.5 ± 2.95	24.4 ± 3.59	2	2	0	2	0	1	NR	NR	15.0 ± 9.3	14.0 ± 7.3
7	Winocour et al. [41]	24.0 ± 3.2	29.8 ± 6.1	NR	NR	0	4	0	1	18	21	60	12

*RALDF* robotic-assisted latissimus dorsi Flap, *RA-DIEP* robotic-assisted deep inferior epigastric perforator flap, *LDF* latissimus dorsi flap, *BMI* body mass index, *Data reported using median and 95% confidence interval, *NR* non-reported

There were 43 and 81 patients with ductal carcinoma in situ among the robotic and conventional surgery groups. Nipple-sparing mastectomy (NSM) was performed among 94 patients subjected to robotic surgery and 97 with conventional surgeries. Immediate robotic breast reconstruction was performed among 200 patients, while 12 received delayed reconstruction. Subsequently, immediate conventional breast reconstruction was performed among 399 patients, whereby 74 patients received delayed reconstruction. Implant-based robotic surgery was conducted among 68 patients, while implant-based conventional surgeries were performed for 80 patients. The quality of the included studies was good, with scores ranging from 66.6% to 83.33% (Table 2).

**Table 2:** Surgery-related data and quality assessment of the included studies

Study ID		ASA score						Tumour pathology					
		I		II		III		DCIS		IDC		Infiltrative other	
		Robotic	Conventional	Robotic	Conventional	Robotic	Conventional	Robotic	Conventional	Robotic	Conventional	Robotic	Conventional
		Number	Number	Number	Number	Number	Number	Number	Number	Number	Number	Number	Number
1	Clemens et al. [36]	NR	NR	NR	NR	NR	NR	0	0	65		11	
2	Eo et al. [42]	NR	NR	NR	NR	NR	NR	4	2	15	18	1	0
3	Houvenaeghel et al. [37]	22	19	23	39	1	1	10	7	24	45	12	7
4	Houvenaeghel et al. [38]	46	24	77	53	3	1	24	10	69	57	30	10
5	Lee et al.	13	114	8	66	0	6	5	52	13	111	3	22
6	Tsai et al. [40]	NR	NR	NR	NR	NR	NR	NR	NR	NR	NR	NR	NR
7	Winocour et al. [41]	NR	NR	NR	NR	NR	NR	NR	NR	NR	NR	NR	NR

Study ID		Type of mastectomy				Timing of breast reconstruction				Number of implants		Quality assessment	
		Nipple sparing		Skin sparing		Immediate		Delayed					
		Robotic	Conventional	Robotic	Conventional	Robotic	Conventional	Robotic	Conventional	Robotic	Conventional		
		Number	Number	Number	Number	Number	Number	Number	Number	Number	Number	%	Decision
1	Clemens et al. [36]	0	0	12	64	0	0	12	64	12	64	66.6%	Good
2	Eo et al. [42]	NR	NR	NR	NR	NR	NR	NR	NR	NR	NR	66.6%	Good
3	Houvenaeghel et al. [37]	0	0	46	59	46	59	0	0	16	7	75%	Good
4	Houvenaeghel et al. [38]	76	5	50	69	126	78	0	0	40	9	75%	Good
5	Lee et al.	18	92	3	94	21	186	0	0	NR	NR	83.33%	Good
6	Tsai et al. [40]	NR	NR	NR	NR	7	76	0	10	NR	NR	66.6%	Good
7	Winocour et al. [41]	NR	NR	NR	NR	NR	NR	NR	NR	NR	NR	75%	Good

*ASA score* the American Society of Anesthesiology Score, *DCIS* ductal carcinoma in situ, *IDC* invasive ductal carcinoma, *NR* non-reported

## Surgical outcomes

### Duration of surgery

**Fig. 2:**
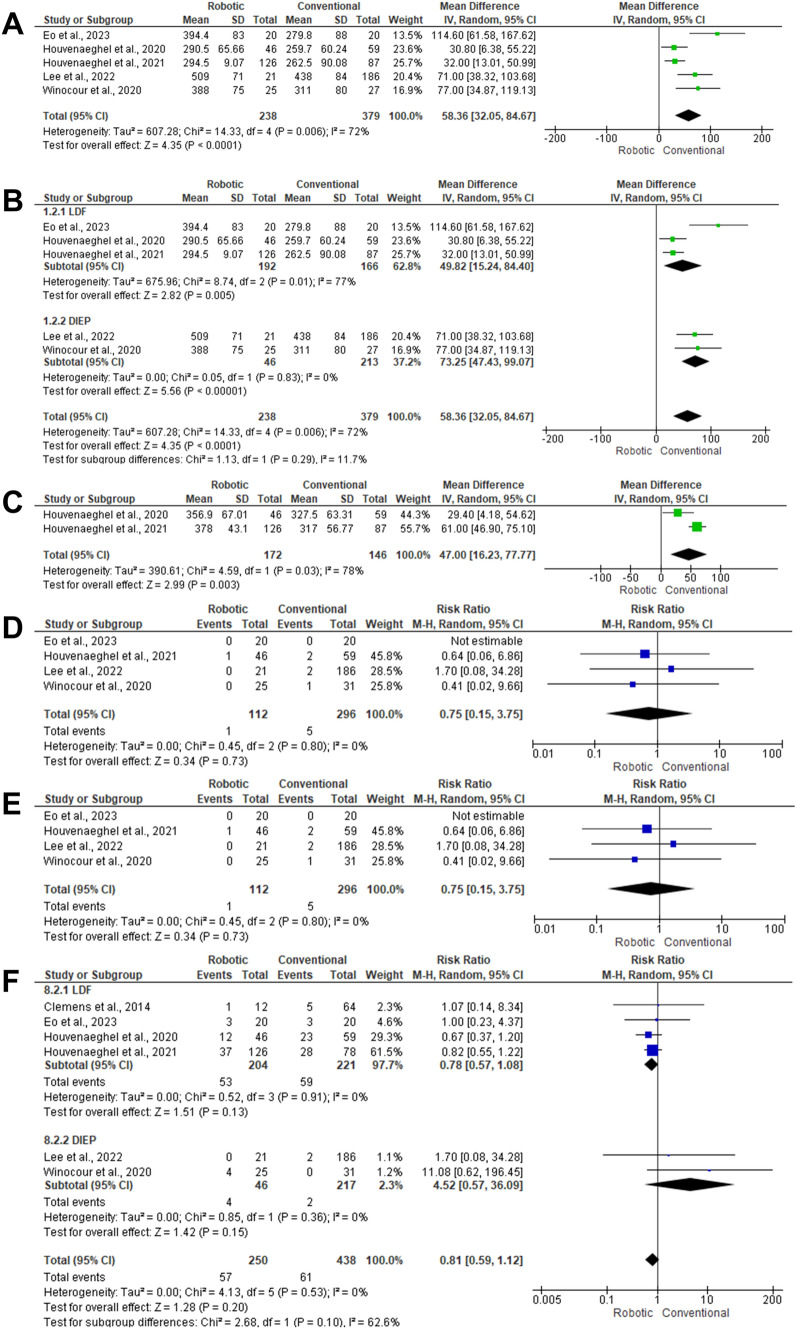
Forest plot of summary analysis of the **(A)** Mean Difference (MD) and 95% CI of mean operative time between robotic and conventional autologous breast reconstruction **(B)** Subgroup analysis of mean Difference (MD) and 95% CI of mean operative time between robotic and conventional autologous breast reconstruction based on the type of reconstruction **(C)** Mean Difference (MD) and 95% CI of mean anesthesia time between robotic and conventional autologous breast reconstruction **(D)** The risk ratio (RR) and 95% confidence intervals of the risk of donor site hematoma between robotic and conventional autologous breast reconstruction. **(E)** The risk ratio (RR) and 95% confidence intervals of the risk of donor site seroma between robotic and conventional autologous breast reconstruction. **(F)** Subgroup analysis of the risk ratio (RR) and 95% confidence intervals of the risk of donor site seroma between robotic and conventional autologous breast reconstruction based on the type of breast reconstruction. Size of the green or blue squares is proportional to the statistical weight of each trial. The grey diamond represents the pooled point estimate. The positioning of both diamonds and squares (along with 95% CIs) beyond the vertical line (unit value) suggests a significant outcome (*IV* inverse variance, *LDF* latissimus dorsi flap, *DIEP* deep inferior epigastric artery perforator flap)

Five articles included 617 patients with autologous breast reconstruction [37–39, 41, 42]. There was a significant prolonged duration of surgery among patients who underwent robotic surgery (MD 58.36; 95% CI 32.05,84.67; *P* < 0.001) with heterogeneity between the included studies (*I*^2^ = 72%, *P* = 0.006). Subgroup analysis based on the type of flaps used for reconstruction was performed. There was a statistically significant prolonged duration of surgery among patients who underwent robotic LDF (MD 49.82; 95% CI 15.24,84.40; *P* < 0.001), with a relatively more prolonged duration of surgery among patients treated with DIEP flap (MD 73.25;95% CI 47.43,99.07; *P* < 0.001) (Fig. 2A, B).

## Duration of anesthesia

The difference in the anaesthesia duration was reported in two articles, including 318 patients [37, 38]. In the random-effects model (*I*^2^ = 78%, *P* = 0.003), there was a statistically significant prolonged duration of anaesthesia among patients who underwent robotic surgery (MD 47; 95% CI 16.23, 77.77; *P* = 0.003) (Fig. 2C).

## Complications

### Donor-site hematoma

Four articles included 408 patients, reported the impact of robotic surgery on the risk of donor site hematoma [38, 39, 41, 42]. There was no statistically significant difference between robotic and conventional surgeries (RR 0.75; 95% CI 0.15,3.75; *P* = 0.73) (Fig. 2D).

### Donor-site seroma

Five articles included 648 patients with autologous breast reconstruction and assessed the impact of robotic surgery on the risk of donor site seroma [36–39, 41]. There was no statistically significant difference between robotic surgery and conventional surgery (MD 0.81; 95% CI 0.59,1.12; *P* = 0.20) in the random-effects model (*I*^2^ = 0%, *P* = 0.35). Subgroup analysis based on the type of reconstruction revealed a relatively high risk of seroma among patients with DIEP flap (MD 4.52; 95% CI 0.57, 36.09) without significant difference (*P* = 0.15) (Fig. 2E, F).

### Donor site infection

**Fig. 3:**
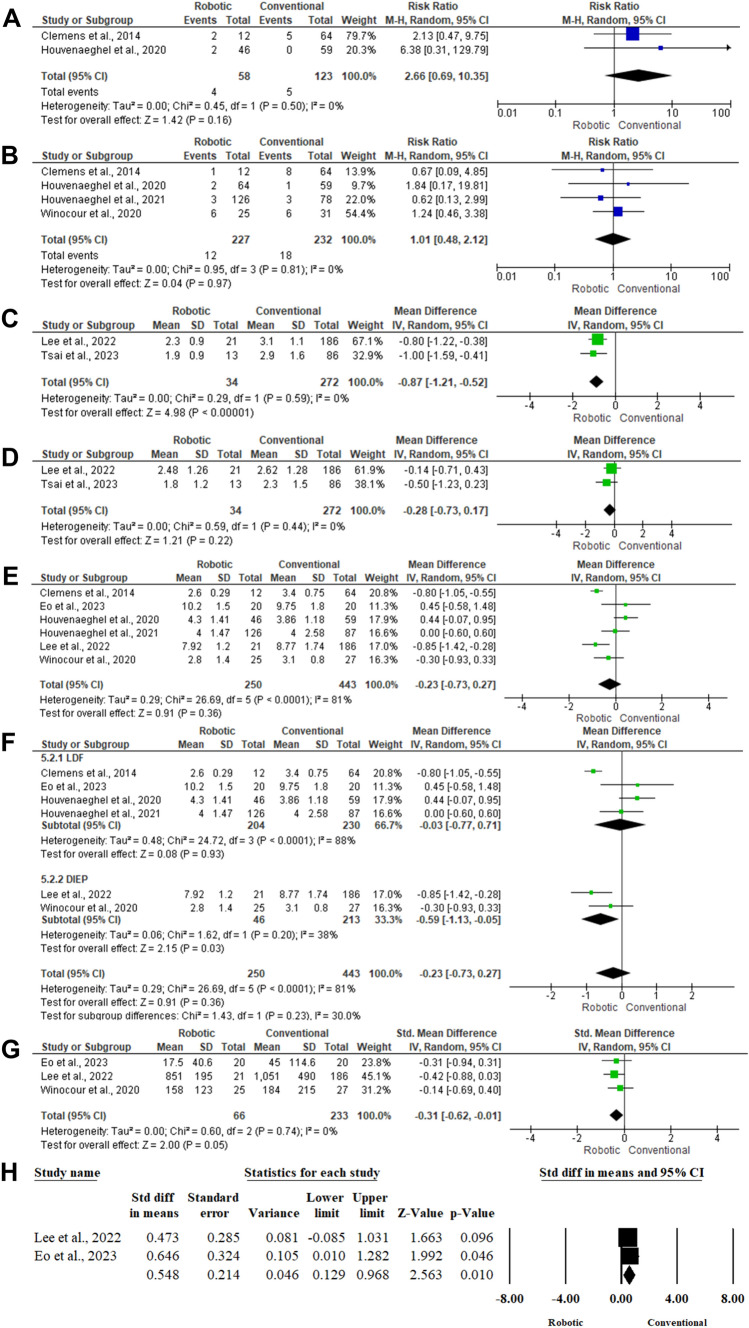
Forest plot of summary analysis of the **(A)** The risk ratio (RR) and 95% confidence intervals of the risk of donor site infection between robotic and conventional autologous breast reconstruction **(B)** The risk ratio (RR) and 95% confidence intervals of the risk of revision surgery between robotic and conventional autologous breast reconstruction **(C)** Mean Difference (MD) and 95% CI of mean pain intensity at the 1st day post-operative between robotic and conventional autologous breast reconstruction **(D)** Mean Difference (MD) and 95% CI of mean pain intensity at the 2nd day post-operative between robotic and conventional autologous breast reconstruction **(E)** Mean Difference (MD) and 95% CI of mean hospital stays between robotic and conventional autologous breast reconstruction **(F)** Subgroup analysis of the mean Difference (MD) and 95% CI of mean hospital stays between robotic and conventional autologous breast reconstruction based on the type of breast reconstruction. **(G)** Mean Difference (MD) and 95% CI of post-operative pain intensity between robotic and conventional autologous breast reconstruction. **(H)** Standardized Mean Difference (SMD) and 95% CI of mean score of satisfaction with breasts between robotic and conventional autologous breast reconstruction Size of the green or blue squares is proportional to the statistical weight of each trial. The grey diamond represents the pooled point estimate. The positioning of both diamonds and squares (along with 95% CIs) beyond the vertical line (unit value) suggests a significant outcome (*IV* inverse variance, *LDF* latissimus dorsi flap, *DIEP* deep inferior epigastric artery perforator flap)

The risk of donor site infection between robotic and conventional techniques was reported in two articles, including 181 patients [36, 37]. There was no statistically significant difference between robotic surgery and conventional surgery (MD 2.66; 95% CI; 0.69,10.35; *P* = 0.16) in the random-effects model (*I*^2^ = 0%, *P* = 0.50) (Fig. 3A).

### Revision rate

The risk of revision surgery between robotic and conventional surgery was reported in four articles [36–38, 41]. There was no statistically significant difference between both groups (RR 1.01;95% CI; 0.48, 2.12; *P* = 0.97) in the random-effects model (*I*^2^ = 0%, *P* = 0.81) (Fig. 3B).

## Functional outcomes

### Pain intensity at 1st day

Two articles included 306 patients with autologous breast reconstruction [39, 40]. The mean level of pain intensity at the 1st day was significantly lower among patients who received robotic breast surgery, in contrast to those who received conventional surgery (MD − 0.87; 95%CI; − 1.21,− 0.52; *P* < 0.001) (Fig. 3C).

### Pain intensity at 2nd day

The mean levels of pain intensity between robotic and conventional surgeries were evaluated among two articles that included 306 patients [39, 40]. There was no statistically significant difference between either group regarding the pain intensity at the 2nd day post-operatively (MD − 0.28;95% CI; − 0.73,0.17; *P* = 0.22) (Fig. 3D).

### Length of hospital stays

Six articles included 693 patients who underwent autologous breast reconstruction [36–39, 41, 42]. There was no statistically significant difference between both groups regarding the mean length of hospital stays (MD − 0.23; 95% CI; − 0.73, 0.27; *P* = 0.36) in the random-effects model (*I*^2^ = 81%, *P* < 0.001). Subgroup analysis based on the type of reconstruction revealed a statistically significant prolonged length of hospitalization among patients with conventional DIEP flap surgery (MD -0.59;95% CI; − 1.13, − 0.05; *P* = 0.03) (Fig. 2F, 3E).

### Post-operative analgesics usage

Three articles [39, 41, 42] included 299 patients, and reported the difference in the post-operative analgesics’ usage between robotic and conventional techniques. There was no statistically significant difference between both groups (MD − 0.31; 95% CI; − 0.62, − 0.01; *P* = 0.05) in the random-effects model (*I*^2^ = 0%, *P* = 0.74) (Fig. 3G).

## Patients’ satisfaction and overall costs

Tsai et al., 2023 reported the mean incision length among patients with DIEP flaps. The mean length of incision was 2.67 ± 1.13 cm among the robotic surgery group, in contrast to 8.14 ± 1.69 cm within the conventional group [40]. Two articles included 247 patients revealed the mean score of satisfaction with breasts between the robotic and conventional techniques [42, 43]. Pooling the data in the random-effects model (*I*^2^ = 0%, *P* = 0.68) revealed a statistically significant more satisfaction with breasts among patients operated with robotic surgery (SMD 0.548; 95% CI;0.129, 0.968; *P* = 0.01). The overall costs of robotic surgery were reported in the Houvenaeghel et al., 2021 study. The costs of the robotic surgery was 10,398 (9875–10,921) US dollars, in contrast to 7788 (7352–8224) within the conventional group [38] (Fig. 3H).

## Discussion

Robotic-assisted surgery has now become an integral part of all surgical specialities. However, there has been a delayed adoption of robotic techniques in the plastic surgery field. This is because of insufficient clinical studies that revealed the feasibility of this technology in different surgical settings [28, 44]. The present systematic review revealed the safety and effectiveness of robotic surgery in autologous breast reconstruction using LDF and DIEP flap. This innovation achieved acceptable surgical and functional outcomes with minimal adverse events. This included less post-operative pain, shorter post-operative hospital stays, and better cosmetic outcomes than the conventional open techniques. There was a similar risk of complications between robotic and conventional autologous breast reconstruction. However, robotic-based autologous breast reconstruction necessitated a prolonged duration of surgery, particularly among patients subordinated to DIEP flap. Robotic technology minimizes human error risk and enhances patients’ safety in autologous breast reconstruction. This reduces the complication risk and promotes a successful long-term surgical and functional outcome.

Robotic-based autologous breast reconstruction is a promising minimally invasive technique. The present meta-analysis revealed better pain control, shorter post-operative hospital stays, and smaller incisions among patients subjected to robotic breast reconstruction. Khan et al., 2022 highlighted the feasibility of robotic harvesting of DIEP flaps without converting to the open technique. This was achieved with minimal complications, shorter post-operative hospital stays, and improved cosmetic outcomes [45]. Consistent with these results, Vourtsis et al., 2022 revealed the safety of robotic harvesting of LDF with excellent aesthetic outcomes, even in the settings of radiotherapy or delayed reconstruction [46]. De la Cruz-Ku et al. reported a significantly lower risk of complications with robotic NSM, yet with prolonged operative time and more lengthy hospital stays [47]. The lesser early post-operative pain with robotic surgery interferes with delayed post-operative recovery and poor outcomes after breast surgeries [48]. Robotic surgery involves minimal tissue handling, less invasiveness and tissue traction, and better surgical exposure. This allowed a safe raising of the harvested flaps with minimal donor site complications and acceptable aesthetic results [25]. In this respect, Chen et al. reported effective breast reconstruction with low complications and better quality of life after robotic-assisted breast surgeries [24]. Roy et al., 2023 reported a comparable complication rate and shorter incision length, yet with prolonged operation time when comparing robotic and traditional autologous breast reconstructive procedures [26].

The robotic-based breast reconstruction surgeries convey significant advantages. The ability to offer enhanced precision and execute fine, delicate movements could improve the outcomes of breast surgeries. The technology provides a clear, detailed view of the surgical field. This accurately aids in identifying vital structures, such as blood vessels and nerves, necessary for harvesting flaps for breast reconstruction [49, 50]. Despite these advantages, the robotic technology has multiple limitations. The acquisition and maintenance of robotic systems represent a substantial burden for healthcare facilities. The present systematic review revealed a relatively higher cost of robotic-based autologous breast reconstruction. The robotic systems require specialized training programs and well-prepared facilities. The training is time-consuming and costly and necessitates dedicated efforts for proficiency. These technical challenges may result in a lengthy learning curve, affecting the integration of robotic technology into the breast surgery practice [51, 52]. The substantial costs and the challenges of robotic technology raise concerns regarding the ability of patients at various healthcare facilities to benefit from the advantages of this promising technology. Further studies are needed to comprehensively evaluate the cost-effectiveness of different robotic platforms for autologous breast reconstruction. This could be evaluated in the context of less post-operative hospital stays and comparable complications to the conventional technique.

In the present study, robotic breast surgery required prolonged operative time. This included a prolonged duration of time after DIEP Flap relative to LDF. Robotic surgery is a complex procedure requiring additional time to prepare the equipment, troubleshooting, highly skillful surgeons, and well-prepared healthcare facilities [53]. Furthermore, the time needed to reach the flap's pedicle, dissection around it, and harvest the flap with robotic surgery is more pronounced than open techniques. This time was even more pronounced during DIEP flap harvesting, even with robotic technologies [45]. The prolonged operative time with robotic surgery may increase the cost of the procedure by approximately 1.5 folds. However, the robotic breast reconstruction costs may be balanced by the resulting satisfactory clinical and surgical outcomes. Parallel with these findings, Nehme et al. reported the prolonged set-up and operating time, demanding learning curve, and high costs with robotic-assisted reconstructive surgeries [28]. In this respect, reconstructive surgeons' tendency to use robotic platforms for breast reconstruction may lead to a considerable decline in the future operative time, decreasing the learning curve and minimizing the overall costs of the procedure [54].

There was a relatively similar risk of complications between robotic and conventional autologous breast reconstruction procedures. These findings were parallel with Filipe et al., 2022 who reported a non-significant difference between robotic and conventional NSM regarding the risk of post-operative complications [55]. Clarke et al. reported a low risk of complications among patients subjected to robotic NSM and immediate breast reconstruction [56]. However, patients subjected to robotic DIEP flaps were at a relatively higher risk of donor site seroma. DIEP flap is one of the most advanced reconstructive procedures, necessitating meticulous harvesting. In the present meta-analysis, the DIEP flap was performed robotically using a transabdominal pre-peritoneal approach. This technique is more invasive than the extra-peritoneal approach, requiring a peritoneal incision to enter the abdominal cavity to reach the vascular pedicle. Subsequently, the pre-peritoneal technique represents a burden for reconstructive surgeons unfamiliar with the abdominal cavity's detailed anatomy. Despite being associated with a substantial risk of complications, no patient experienced abdominal hernia, bowel perforation, or intra-abdominal bleeding in the present meta-analysis. This is because the robotic platform allowed the surgeons to harvest the vascular pedicle of the DIEP flap using minor fascial defects [57]. Furthermore, the platform allowed the operator to follow the vascular pedicle in an inside-out fashion, limiting the dissection through the abdominal muscles and the neurovascular plane [58]. Multiport robotic surgery necessitates multiple openings in a narrow pre-peritoneal space, bearing a substantial risk of injury to the neighboring tissues and bowel perforation. Single-port robotic breast reconstruction can reduce the risk of intra-abdominal complications in which the movement can be executed without collision between the robotic arms. Extra-peritoneal robotic harvesting of the DIEP flaps could minimize the risk of fascial incisions and the damage encountered to the motor nerves and rectus muscle with the pre-peritoneal approach. However, it is associated with prolonged operating time and a challenging learning curve compared to the pre-peritoneal approach [59, 60].

This meta-analysis gathered evidence related to the effectiveness of robotic-assisted autologous breast reconstruction. However, the study's results should be evaluated in the context of some limitations. All the included studies were observational, with four articles of retrospective design. This conveys a higher risk of information selection bias and reporting bias. Furthermore, most of the included studies included a relatively small number of patients subjected to robotic surgery. There was statistically significant heterogeneity between the included studies. Such heterogeneity may reveal the difference in the surgical procedures, demographic characteristics, study designs, or follow-up periods. Prospective randomized clinical trials with adequate sample sizes and prolonged post-operative follow-up protocols are required to mitigate the limitations of the included observational studies.

## Conclusions

The robotic technology marks a transformative innovation in breast reconstruction. The present meta-analysis revealed the feasibility, safety, and effectiveness of robotic flap harvesting for breast reconstruction. The robotics allowed a successful LDF and DIEP flap harvesting with acceptable surgical and functional outcomes. Robotic breast reconstruction was associated with less post-operative pain, and shorter post-operative hospital stays with a comparable risk of complications to the conventional techniques. Despite these promising advantages, robotic surgery conveys substantial challenges, including prolonged operative time, high costs, and specialized, well-prepared healthcare facilities.

## Supplementary Information

Below is the link to the electronic supplementary material.

Supplementary file1 (DOCX 30 KB)

## Data Availability

The datasets used in the present study are available from the first author and corresponding authors on reasonable request.

## Abbreviations

DIEP flap: Deep inferior epigastric artery perforator flap
LDF: Latissimus dorsi flap
PRISMA: Preferred reporting items for systematic reviews and meta-analysis
NSM: Nipple-sparing mastectomy

## References

[1] Zhu JW, Charkhchi P, Adekunte S, Akbari MR (2023) What Is Known about Breast Cancer in Young Women? Cancers. 10.3390/cancers15061917 36980802 10.3390/cancers15061917 PMC10047861

[2] Giaquinto AN, Sung H, Miller KD, Kramer JL, Newman LA, Minihan A, Jemal A, Siegel RL (2022) Breast cancer statistics. CA Cancer J Clin 72(6):524–541. 10.3322/caac.21754 36190501 10.3322/caac.21754

[3] Sung H, Ferlay J, Siegel RL, Laversanne M, Soerjomataram I, Jemal A, Bray F (2021) Global cancer statistics 2020: GLOBOCAN estimates of incidence and mortality worldwide for 36 cancers in 185 countries. CA cancer J Clin 71(3):209–249. 10.3322/caac.21660 33538338 10.3322/caac.21660

[4] Fanakidou I, Zyga S, Alikari V, Tsironi M, Stathoulis J, Theofilou P (2018) Mental health, loneliness, and illness perception outcomes in quality of life among young breast cancer patients after mastectomy: the role of breast reconstruction. Qual Life Res 27:539–543 29119452 10.1007/s11136-017-1735-x

[5] Deshpande V, Shinde RK, Deo D, Hippargekar P, Venurkar SV, Deshpande VP (2022) Assessment of quality of life in patients of mastectomy with chemotherapy. Cureus J Med. 10.7759/cureus.27703 10.7759/cureus.27703 PMC9440994 36081965

[6] Shaterian A, Gandy J, Lalezari S, Smith S, Paydar K (2016) Patient race and provider predict patient satisfaction following post-mastectomy breast reconstruction. World J Plast Surg 5(2):114 27579266 PMC5003946

[7] Rautalin M, Jahkola T, Roine RP (2022) Breast reconstruction–prospective follow up on breast cancer patients’ health-related quality of life. World J Surg. 10.1007/s00268-021-06426-4 35001140 10.1007/s00268-021-06426-4 PMC8885544

[8] Jonczyk MM, Jean J, Graham R, Chatterjee A (2019) Surgical trends in breast cancer: a rise in novel operative treatment options over a 12 year analysis. Breast cancer Res Treat 173:267–274 30361873 10.1007/s10549-018-5018-1 PMC6486837

[9] Statistics P (2018) American Society of Plastic Surgeons. 2018 Plastic Surgery Statistics Report. Plast Surg 25. https://www.plasticsurgery.org/documents/News/Statistics/2018/plastic-surgery-statistics-full-report-2018.pdf

[10] Pusic AL, Matros E, Fine N, Buchel E, Gordillo GM, Hamill JB, Kim HM, Qi J, Albornoz C, Klassen AF, Wilkins EG (2017) Patient-reported outcomes 1 year after immediate breast reconstruction: results of the mastectomy reconstruction outcomes consortium study. J Clin Oncol 35(22):2499–2506. 10.1200/JCO.2016.69.9561 28346808 10.1200/JCO.2016.69.9561 PMC5536162

[11] Sood R, Easow JM, Konopka G, Panthaki ZJ (2018) Latissimus dorsi flap in breast reconstruction: recent innovations in the workhorse flap. Cancer Control 25(1):1073274817744638 29334788 10.1177/1073274817744638 PMC5933575

[12] Macadam SA, Bovill ES, Buchel EW, Lennox PA (2017) Evidence-based medicine: autologous breast reconstruction. Plast Reconstrct Surg 139(1):204e-e229 10.1097/PRS.0000000000002855 28027256

[13] Opsomer D, Van Landuyt K (2018) Indications and controversies for nonabdominally-based complete autologous tissue breast reconstruction. Clin Plast Surg 45(1):93–100 29080664 10.1016/j.cps.2017.08.012

[14] Mericli AF, Szpalski C, Schaverien MV, Selber JC, Adelman DM, Garvey PB, Villa MT, Robb G, Baumann DP (2019) The latissimus dorsi myocutaneous flap is a safe and effective method of partial breast reconstruction in the setting of breast-conserving therapy. Plast Reconstrct Surg 143(5):927e–935e. 10.1097/PRS.0000000000005577 10.1097/PRS.0000000000005577 31033814

[15] Chang EI, Chang EI, Soto-Miranda MA, Zhang H, Nosrati N, Robb GL, Chang DW (2013) Comprehensive analysis of donor-site morbidity in abdominally based free flap breast reconstruction. Plast Reconstrct Surg 132(6):1383–1391 10.1097/PRS.0b013e3182a805a3 24005365

[16] Fauconnier M, Burnier P, Jankowski C, Loustalot C, Coutant C, Vincent L (2022) Comparison of postoperative complications following conventional latissimus dorsi flap versus muscle-sparing latissimus dorsi flap breast reconstruction. J Plast Reconstruct Aesthetic Surg 75(10):3653–3663 10.1016/j.bjps.2022.06.084 36100540

[17] DellaCroce FJ, DellaCroce HC, Blum CA, Sullivan SK, Trahan CG, Wise MW et al (2019) Myth-busting the DIEP flap and an introduction to the abdominal perforator exchange (APEX) breast reconstruction technique: a single-surgeon retrospective review. Plast Reconstrct Surg. 143(4):992. 10.1097/PRS.0000000000005484 10.1097/PRS.0000000000005484 PMC6445603 30730497

[18] Hivelin M, Soprani A, Schaffer N, Hans S, Lantieri L (2018) Minimally invasive laparoscopically dissected deep inferior epigastric artery perforator flap: an anatomical feasibility study and a first clinical case. Plast Reconstrct Surg 141(1):33–39 10.1097/PRS.0000000000003989 28915211

[19] Selber JC, Baumann DP, Holsinger CF (2012) Robotic harvest of the latissimus dorsi muscle: laboratory and clinical experience. J Reconstruct Microsurg 28(07):457–464 10.1055/s-0032-1315789 22744894

[20] Lai HW, Lin SL, Chen ST, Lin YL, Chen DR, Pai SS, Kuo SJ (2018) Robotic nipple sparing mastectomy and immediate breast reconstruction with robotic latissimus dorsi flap harvest–technique and preliminary results. J of Plast Reconstruct Aesthetic Surg 71(10):e59–e61 10.1016/j.bjps.2018.07.006 30122600

[21] Selber JC (2020) The robotic DIEP flap. Pl Plast Reconstrct Surg 145(2):340–343 10.1097/PRS.0000000000006529 31985617

[22] Selber JC, Baumann DP, Holsinger FC (2012) Robotic latissimus dorsi muscle harvest: a case series. Plast Reconstrct Surg 129(6):1305–1312 10.1097/PRS.0b013e31824ecc0b 22634647

[23] Chung J-H, You H-J, Kim H-S, Lee B-I, Park S-H, Yoon E-S (2015) A novel technique for robot assisted latissimus dorsi flap harvest. J Plast Reconstruct & Aesthetic Surg 68(7):966–972 10.1016/j.bjps.2015.03.021 25886882

[24] Chen K, Zhang J, Beeraka NM, Sinelnikov MY, Zhang X, Cao Y, Lu P (2022) Robot-Assisted Minimally Invasive Breast Surgery: Recent Evidence with Comparative Clinical Outcomes. J of Clin Med. 10.3390/jcm11071827 10.3390/jcm11071827 PMC8999956 35407434

[25] Bishop SN, Selber JC (2021) Minimally invasive robotic breast reconstruction surgery. Gland Surg 10(1):469 33634004 10.21037/gs-20-248 PMC7882313

[26] Roy N, Alessandro CJ, Ibelli TJ, Akhavan AA, Sharaf JM, Rabinovitch D, Henderson PW, Yao A (2023) The expanding utility of robotic-assisted flap harvest in autologous breast reconstruction: a systematic review. J of Clin Med. 12(15):4951. 10.3390/jcm12154951 37568353 10.3390/jcm12154951 PMC10419897

[27] Jain Y, Lanjewar R, Shinde RK (2024) Revolutionising breast surgery: a comprehensive review of robotic innovations in breast surgery and reconstruction. Cureus. 10.7759/cureus.52695 38384645 10.7759/cureus.52695 PMC10879655

[28] Nehme N, J, Neville JJ, Bahsoun AN, (2017) The use of robotics in plastic and reconstructive surgery: a systematic review. JPRAS Open 13:1–10

[29] Collaboration C (2008) Cochrane handbook for systematic reviews of interventions: Cochrane Collaboration. https://onlinelibrary.wiley.com/doi/pdf/10.1002/9780470712184

[30] Moher D, Liberati A, Tetzlaff J, Altman DG (2009) Preferred reporting items for systematic reviews and meta-analyses: the PRISMA statement. BMJ 339:b2535. 10.1136/bmj.b2535 10.1136/bmj.b2535 PMC2714657 19622551

[31] Rohatgi A (2021) WebPlotDigitizer. https://automeris.io. Accessed 20 Nov 2021

[32] National Heart L, Institute B (2014) National institute of health, quality assessment tool for observational cohort and cross-sectional studies. National Heart. Lung, and Blood Institute, Bethesda

[33] Hozo SP, Djulbegovic B, Hozo I (2005) Estimating the mean and variance from the median, range, and the size of a sample. BMC Med Res Methdol 5(1):13 10.1186/1471-2288-5-13 PMC1097734 15840177

[34] Higgins JP, Thompson SG, Deeks JJ, Altman DG (2003) Measuring inconsistency in meta-analyses. BMJ 327(7414):557 12958120 10.1136/bmj.327.7414.557 PMC192859

[35] Borenstein M (2022) Comprehensive meta-analysis software. Systematic reviews in health research: meta-analysis in contex. Wiley, pp 535–48

[36] Clemens MW, Kronowitz S, Selber JC (2014) Robotic-assisted latissimus dorsi harvest in delayed-immediate breast reconstruction. Semin Plast Surg 28(01):020–025 10.1055/s-0034-1368163 PMC3946018 24872775

[37] Houvenaeghel G, El Hajj H, Schmitt A, Cohen M, Rua S, Barrou J, Lambaudie E, Bannier M (2020) Robotic-assisted skin sparing mastectomy and immediate reconstruction using latissimus dorsi flap a new effective and safe technique: a comparative study. Surg Oncol 1(35):406–411 10.1016/j.suronc.2020.09.022 33035789

[38] Houvenaeghel G, Rua S, Barrou J, Troy AV, Knight S, Cohen M, Bannier M (2021) Robotic versus conventional latissimus dorsi-flap harvested for immediate breast reconstruction. J Surg Res 4(4):749–764

[39] Lee MJ, Won J, Song SY, Park HS, Kim JY, Shin HJ, Kwon YI, Lee DW, Kim NY (2022) Clinical outcomes following robotic versus conventional DIEP flap in breast reconstruction: a retrospective matched study. Frontiers in Oncol 14(12):989231 10.3389/fonc.2022.989231 PMC9515388 36185209

[40] Tsai CY, Kim BS, Kuo WL, Liu KH, Chang TN, Cheong DC, Huang JJ (2023) Novel port placement in robot-assisted DIEP flap harvest improves visibility and bilateral diep access: early controlled cohort study. Plast Reconstrct Surg 152(4):590e-e595 10.1097/PRS.0000000000010470 36995211

[41] Winocour S, Tarassoli S, Chu CK, Liu J, Clemens MW, Selber JC (2020) Comparing outcomes of robotically assisted latissimus dorsi harvest to the traditional open approach in breast reconstruction. Plast Reconstrct Surg 146(6):1221–1225 10.1097/PRS.0000000000007368 33234946

[42] Eo PS, Kim H, Lee JS, Lee J, Park HY, Yang JD (2023) Robotic-assisted latissimus dorsi flap harvest in partial breast reconstruction: comparison with endoscopic and conventional approaches. Aesthetic Surg J 44(1):38–46 10.1093/asj/sjad280 37610290

[43] Lee MJ, Won J, Song SY, Park HS, Kim JY, Shin HJ, Kwon YI, Lee DW, Kim NY (2022) Clinical outcomes following robotic versus conventional DIEP flap in breast reconstruction: a retrospective matched study. Front Oncol 14(12):989231 10.3389/fonc.2022.989231 PMC9515388 36185209

[44] Selber JC (2017) Can I make robotic surgery make sense in my practice? Plast and Reconstrct Surg 139(3):781e-e792 10.1097/PRS.0000000000003151 28234863

[45] Khan MT, Won BW, Baumgardner K, Lue M, Montorfano L, Hosein RC, Wang HT, Martinez RA (2022) Literature review: robotic-assisted harvest of deep inferior epigastric flap for breast reconstruction. Ann Plst Surg 89(6):703–708 10.1097/SAP.0000000000003326 36416707

[46] Vourtsis SA, Paspala A, Lykoudis PM, Spartalis E, Tsourouflis G, Dimitroulis D, Pikoulis E, Nikiteas N (2021) Robotic-assisted harvest of latissimus dorsi muscle flap for breast reconstruction: review of the literature. J Rob Surg 23:1–5 10.1007/s11701-021-01232-5 33755925

[47] De la Cruz-Ku G, Chambergo-Michilot D, Perez A, Valcarcel B, Pamen L, Linshaw D, Chatterjee A, LaFemina J, Boughey JC (2023) Outcomes of robotic nipple-sparing mastectomy versus conventional nipple-sparing mastectomy in women with breast cancer: a systematic review and meta-analysis. J Rob Surg 20:1–7 10.1007/s11701-023-01547-5 36808041

[48] Tan YY, Liaw F, Warner R, Myers S, Ghanem A (2019) Enhanced recovery pathways for flap-based reconstruction: systematic review and meta-analysis. Aesthetic Plast Surg 1:1–20 10.1007/s00266-021-02233-3 33821314

[49] Sayari AJ, Pardo C, Basques BA, Colman MW (2019) Review of robotic-assisted surgery: what the future looks like through a spine oncology lens. Ann transl Med. 10.21037/atm.2019.04.69 31297389 10.21037/atm.2019.04.69 PMC6595200

[50] Ma L, Fei B (2021) Comprehensive review of surgical microscopes: technology development and medical applications. J Biomed Opt 26(1):010901 33398948 10.1117/1.JBO.26.1.010901 PMC7780882

[51] Lawrie L, Gillies K, Duncan E, Davies L, Beard D, Campbell MK (2022) Barriers and enablers to the effective implementation of robotic assisted surgery. PLoS ONE 17(8):e0273696 36037179 10.1371/journal.pone.0273696 PMC9423619

[52] Sridhar AN, Briggs TP, Kelly JD, Nathan S (2017) Training in robotic surgery—an overview. Current Urol reports 18:1–8 10.1007/s11934-017-0710-y PMC5486586 28647793

[53] Haidegger T (2019) Autonomy for surgical robots: Concepts and paradigms. IEEE Trans Med Robot Bionics 1(2):65–76

[54] Peteoaca A, Istrate A, Tanase A, Mocanu J, MICSA C, Ionita L, (2018) A review of robotic surgery evolution, current applications and future prospects. Sci Works Series C, Vet Med 64(2):59–69

[55] Filipe MD, de Bock E, Postma EL, Bastian OW, Schellekens PP, Vriens MR, Witkamp AJ, Richir MC (2022) Robotic nipple-sparing mastectomy complication rate compared to traditional nipple-sparing mastectomy: a systematic review and meta-analysis. J Robot Surg 16(2):265–272 34128142 10.1007/s11701-021-01265-w PMC8960562

[56] Clarke P, de Miranda PD, de Sá NC, Cavalcante JM, de Oliveira F (2020) Robotic breast surgery: the pursue for excellence in treatment and satisfaction–a review. Mastol 30:1–7

[57] Manrique OJ, Bustos SS, Mohan AT, Nguyen MD, Martinez-Jorge J, Forte AJ, Terzic A (2020) Robotic-assisted DIEP flap harvest for autologous breast reconstruction: a comparative feasibility study on a cadaveric model. J Reconstrct Microsurg 36(05):362–368 10.1055/s-0040-1701666 32106313

[58] Daar DA, Anzai LM, Vranis NM, Schulster ML, Frey JD, Jun M, Zhao LC, Levine JP (2022) Robotic deep inferior epigastric perforator flap harvest in breast reconstruction. Microsurg 42(4):319–325 10.1002/micr.30856 34984741

[59] Choi JH, Song SY, Park HS, Kim CH, Kim JY, Lew DH, Roh TS, Lee DW (2021) Robotic DIEP flap harvest through a totally extraperitoneal approach using a single-port surgical robotic system. Plast Reconstruct Surg 148(2):307–307. 10.1097/PRS.0000000000008181 10.1097/PRS.0000000000008181 34398082

[60] Wittesaele W, Vandevoort M (2022) Implementing the Robotic deep inferior epigastric perforator Flap in daily practice: a series of 10 cases. J Plast Reconstr Aesthet Surg 75(8):2577–2583 35400592 10.1016/j.bjps.2022.02.054

